# A Novel Non-Digestible, Carrot-Derived Polysaccharide (cRG-I) Selectively Modulates the Human Gut Microbiota while Promoting Gut Barrier Integrity: An Integrated In Vitro Approach

**DOI:** 10.3390/nu12071917

**Published:** 2020-06-29

**Authors:** Pieter Van den Abbeele, Lynn Verstrepen, Jonas Ghyselinck, Ruud Albers, Massimo Marzorati, Annick Mercenier

**Affiliations:** 1 ProDigest BV, Technologiepark 82, 9052 Ghent, Belgium; Pieter.VandenAbbeele@prodigest.eu (P.V.d.A.); lynn.verstrepen@prodigest.eu (L.V.); Jonas.Ghyselinck@prodigest.eu (J.G.); massimo.marzorati@prodigest.eu (M.M.); 2 Nutrileads BV, Bronland 12-N, 6708WH Wageningen, The Netherlands; ruud.albers@nutrileads.com; 3 Center of Microbial Ecology and Technology (CMET), Ghent University, Coupure Links 653, 9000 Ghent, Belgium

**Keywords:** prebiotic, microbiome, SCFA, colon, bifidobacteria, pectin, rhamnogalacturonan, transepithelial electrical resistance (TEER)

## Abstract

Modulation of the gut microbiome as a means to improve human health has recently gained increasing interest. In this study, it was investigated whether cRG-I, a carrot-derived pectic polysaccharide, enriched in rhamnogalacturonan-I (RG-I) classifies as a potential prebiotic ingredient using novel in vitro models. First, digestion methods involving α-amylase/brush border enzymes demonstrated the non-digestibility of cRG-I by host-derived enzymes versus digestible (starch/maltose) and non-digestible controls (inulin). Then, a recently developed short-term (48 h) colonic incubation strategy was applied and revealed that cRG-I fermentation increased levels of health-promoting short-chain fatty acids (SCFA; mainly acetate and propionate) and lactate comparable but not identical to the reference prebiotic inulin. Upon upgrading this fermentation model by inclusion of a simulated mucosal environment while applying quantitative 16S-targeted Illumina sequencing, cRG-I was additionally shown to specifically stimulate operational taxonomic units (OTUs) related to health-associated species such as *Bifidobacterium longum*, *Bifidobacterium adolescentis*, *Bacteroides dorei*, *Bacteroides ovatus*, *Roseburia hominis*, *Faecalibacterium prausnitzii*, and *Eubacterium hallii*. Finally, in a novel model to assess host–microbe interactions (Caco-2/peripheral blood mononuclear cells (PBMC) co-culture) fermented cRG-I increased barrier integrity while decreasing markers for inflammation. In conclusion, by using novel in vitro models, cRG-I was identified as a promising prebiotic candidate to proceed to clinical studies.

## 1. Introduction

The colon contains a vast number of bacteria that largely impact human health. Next to preventing pathogen colonization through secretion of antimicrobial agents [1,2,3], the gut microbiota is involved in food processing, synthesis of essential vitamins and production of health-promoting short-chain fatty acids (SCFA), including acetate, propionate and butyrate [4], upon anaerobic fermentation of for instance dietary fibers [5]. While butyrate is an important energy source for colonocytes with anti-inflammatory and intestinal barrier-protecting effects, propionate exerts anti-lipogenic and cholesterol-lowering effects in the liver [6]. In addition, as with butyrate, propionate has been reported to exert anti-cancer effects in the colon [7,8]. Finally, acetate is used in the liver as a substrate for cholesterol and fatty acid synthesis [9,10]. In terms of composition, the human gut microbiome mainly consists of the *Firmicutes*, *Bacteroidetes*, *Actinobacteria*, *Proteobacteria*, *Fusobacteria*, and *Verrucomicrobia* phyla [11]. Despite having provided key insights, many studies have been limited to (descriptive) analysis of fecal samples as in situ samples from the site of fermentation are difficult to obtain. To allow in-depth research focusing on not only luminal, but also gut wall-associated mucosal microbes, a novel in vitro model (M-SHIME^®^; Mucosal Simulator of the Human Intestinal Microbial Ecosystem) was recently developed as a complementary in vitro tool [12]. In this model, the mucosal microbiota was enriched with butyrate-producing *Clostridium* cluster XIVa members, correlating with in vivo findings from biopsies [13,14,15,16,17,18].

Modulation of the human gut microbiome as a route to improve human health has gained a lot of interest over recent years. Prebiotics are defined as non-digestible food ingredients that selectively stimulate health-promoting bacteria [19]. A key feature of prebiotics is their resistance to upper gastro-intestinal digestion so that they reach the colon where they are fermented by the gut microbiome. To assess potential digestibility of polysaccharides, two complementary enzyme sources are to be considered, i.e., amylases [20] and brush border enzymes [21]. α-amylase is present in both saliva and pancreatic juice and can liberate maltose from starch [20]. Furthermore, sucrase-isomaltase and maltase-glycoamylase, collectively known as α-glucosidases, are complexes consisting of 4 enzymes that release glucose from oligosaccharides present at the intestinal brush border [21]. These host-derived enzymes jointly digest carbohydrates and hence their specificity determines whether carbohydrates reach the colon and can exert prebiotic effects. Although critical to the definition of potential prebiotic ingredients, studies confirming their indigestibility are scarce.

Fructans, such as fructooligosaccharides (FOS) and inulin are considered to be “gold standard” prebiotics, with human clinical trials supporting their beneficial effect in acute and chronic diseases such as obesity and type 2 diabetes (T2D), allergy, inflammatory bowel disease (IBD), Traveler’s diarrhea and constipation (an overview is given in [19]). As many health-related species belong to the *Bifidobacteriaceae*, prebiotic potential has often been related to an increase of this family. There is however increasing understanding that prebiotics can be fermented by a wider range of gut microbes. Inulin can e.g., also be rapidly fermented by health-promoting *Bacteroidaceae* members, such as *Bacteroides caccae* [22,23]. Currently, there is growing interest to develop novel prebiotics. A specific class of candidates includes pectin-derived polysaccharides enriched in the branched part of pectin, i.e., the rhamnogalacturonan-I (RG-I) domains, which can be extracted from several food crops including carrot. The backbone of RG-I is a repeating unit of the disaccharide [-2)-α-L-rhamnose-(1,4)-α-D-galacturonic acid-(1] and RG-I side-chains consist of galactans (β-1,4-D-galactose (D-Gal) units) and/or arabinans (α-1,5-linked L-arabinofuranose (L-Araf) units with additional L-Araf side-chains), with varying length and composition [24]. Given their structural complexity, RG-I extracts would require fermentation by a consortium of gut microbes with complementary metabolic capabilities [25].

Gut microbial modulation is linked to human health with the concept of a “leaky gut” having gained attention, not only in the context of inflammatory bowel disease, but also in a wider range of psychological and metabolic disorders [26]. Increased intestinal epithelial permeability would allow translocation of bacterial cell wall components, metabolites, or even whole bacteria into the systemic circulation, hence contributing to inflammation and injury, not only in the gut but also in remote organs such as the liver and the brain. Host–microbe interaction studies are increasingly being performed to document this. As an example, microbial fermentation samples can be combined with a human co-culture model [27], including intestinal epithelial cells (Caco-2) and monocytes (THP-1) [28], which demonstrated the gut barrier protective effects together with immuno-modulatory capacity of several prebiotics, including arabinoxylo-oligosaccharides (AXOS) [29], inulin, FOS [30] and a dried yeast fermentate [31]. Despite its usefulness, this model has the limitation that monocytes are only one of the cell lineages involved in the immune response. Therefore, using a co-culture model including Caco-2 cells and peripheral blood mononuclear cells (PBMCs), containing lymphocytes (T-cells, B-cells, and NK-cells), monocytes and dendritic cells [32], could increase the in vivo relevance.

**Figure 1:**
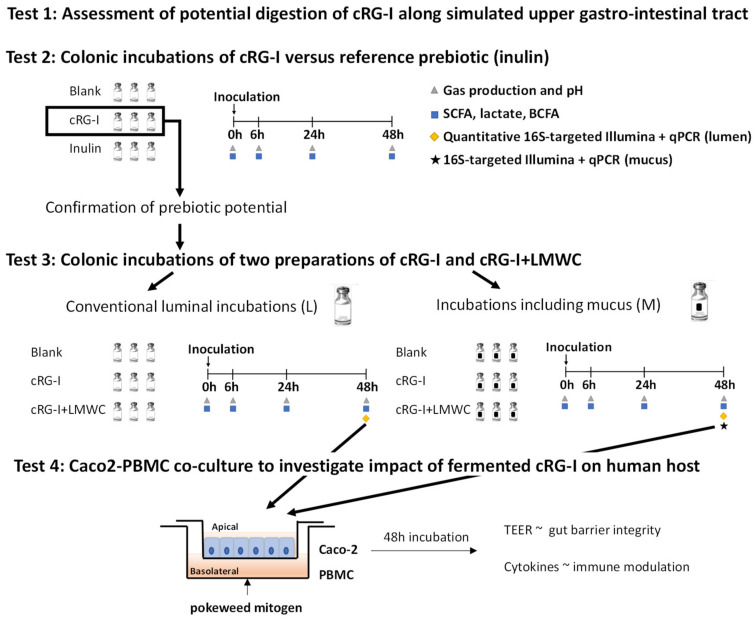
Schematic representation of the integrated in vitro approach to investigate the prebiotic potential of cRG-I (carrot-derived rhamnogalacturonan-I). First, potential digestion of cRG-I by amylase/brush border enzymes was investigated (Test 1). In Test 2, short-term colonic batch incubations were used to assess the prebiotic potential on microbial metabolic activity of cRG-I compared to inulin. In Test 3, the prebiotic potential of two formulations that differed in absence (cRG-I) or presence of low molecular weight carbohydrates (cRG-I+LMWC) was assessed in both conventional luminal incubations (L) and incubations including a mucosal compartment (M). Samples were collected to evaluate the effect of the test products on microbial activity, community composition and on intestinal permeability and immunity (Test 4, Caco-2/PBMC co-culture model).

Therefore, the present study investigated whether a carrot-derived RG-I enriched extract (cRG-I) classifies as a potential prebiotic ingredient using a combination of novel in vitro models (Figure 1). First, potential digestion by amylase/brush border enzymes was investigated (Test 1). Then, a recently developed short-term colonic incubation strategy was applied [29] to assess the potential impact of cRG-I on microbial metabolic activity including inulin as a reference (Test 2). Subsequently, after upgrading the fermentation model by inclusion of a simulated mucosal microbiota [12], more in-depth fermentation tests were performed to characterize the prebiotic potential of two carrot RG-I formulations that differed in absence (cRG-I) or presence of low molecular weight carbohydrates (cRG-I+LMWC) (Test 3). To obtain detailed insights in modulation of microbial composition, a novel technique was used where flow cytometry was combined with 16S-targeted Illumina sequencing to obtain quantitative information at high phylogenetic resolution [33]. Finally, fermentation samples were screened for potential beneficial effects on gut barrier integrity and immune modulation in a newly optimized Caco-2/PBMC co-culture model (test 4).

## 2. Materials and Methods

### 2.1. Products

The two carrot RG-I preparations used in this study (cRG-I [34] and cRG-I+LMWC) were provided by NutriLeads (Wageningen, The Netherlands). Both are pectin-derived polysaccharides and cRG-I+LMWC differs from cRG-I by containing small size sugars, mainly mono- and disaccharides of galactose, glucose, and uronic acids. Pectin is a linear homogalacturonan (HG) interspaced with branched rhamnogalacturonan (RG) regions. HG consists of α-1,4-linked D-galacturonic acid (GalA) monomers while the RG-I backbone is a repeating unit of the disaccharide [-2)-α-L-rhamnose-(1,4)-α-D-galacturonic acid-(1]. The RG-I backbone is decorated with galactans (β-1,4-D-galactose (D-Gal) units) and/or arabinans (α-1,5-linked L-arabinofuranose (L-Araf) units [25]. Inulin (Orafti^®^ HP, 100% inulin, 0% sweetness level, average DP ≥ 23) was generously provided by Beneo GmbH (Mannheim, Germany).

### 2.2. Digestion by Amylase and Brush Border Enzymes (Test 1)

Digestion with amylase was performed as described previously [20]. Briefly, cooked starch (positive control), inulin (negative control) and cRG-I were suspended in distilled water at 15 g/L. A stock solution of 1500U α-amylase/mL (10080, Sigma–Aldrich, Bornem, Belgium) was prepared and added to the substrates to simulate the small intestinal phase, while respecting the ratio of units of amylase versus amount of test product according to the Infogest consensus method (1300 units per gram test product [20]). Samples were incubated for 60′ at 37 °C. Furthermore, digestion with brush border enzymes was performed as previously described in [21]. Briefly, intestinal aceton powder from rat (Sigma–Aldrich, Bornem, Belgium) was dissolved in 0.9% NaCl solution, vortexed, and sonicated. 15 g/L stock solutions of inulin (negative control) and maltose (positive control) were prepared in sodium phosphate buffer (pH 7), while cRG-I was prepared in distilled water. 100 µL enzyme solution and 50 µL substrates were mixed with 100 µL phosphate buffer and incubated for 90′ at 37 °C. High Performance Anion Exchange Chromatography with Pulsed Amperometric Detection (HPAEC-PAD) was used to measure rhamnose, arabinose, galactose, fructose, glucose, maltose, and galacturonic acid in both digestion experiments. All tests were done in technical triplicate.

### 2.3. Short-Term Colonic Batch Incubations (Tests 2 and 3)

Short-term colonic incubations were performed as described in [29]. Briefly, freshly collected fecal material of a healthy human donor (f, 26) was collected and after preparation of an anaerobic fecal slurry inoculated at 10 vol% in a sugar-depleted nutritional medium containing 5.2 g/L K_2_HPO_4_, 16.3 g/L KH_2_PO_4_, 2.0 g/L NaHCO_3_ (Chem-lab NV, Zedelgem, Belgium), 2.0 g/L Yeast Extract, 2.0 g/L pepton (Oxoid, Aalst, Belgium), 1.0 g/L mucin (Carl Roth, Karlsruhe, Germany), 0.5 g/L L-cystein and 2.0 mL/L Tween80 (Sigma–Aldrich, Bornem, Belgium). When mucin-coated carriers were added to the reactors during Test 3, 1.0 g/L mucin was omitted from the nutritional medium. Five mucin-coated carriers were added per reactor after being prepared according to Van den Abbeele et al. (2013) [12]. Test products were dosed at 5 g/L and reactors were anaerobically incubated at 37 °C for 48 h. All experiments were performed in technical triplicate.

### 2.4. Microbial Metabolic Activity (Tests 2 and 3)

Samples were collected upon 0 h, 6 h, 24 h, and 48 h of incubation from each colon reactor. Gas production was measure with a pressure meter (Hand-held pressure indicator CPH6200; Wika, Echt, The Netherlands) and pH measurements were performed with a Senseline pH meter F410 (ProSense, Oosterhout, The Netherlands). Total SCFA were determined as the sum of acetate, propionate, butyrate and branched-chain fatty acids (bCFA; isobutyrate, isovalerate and isocaproate) levels, and were measured as described previously [35]. Lactate production was assessed with a commercially available kit (R-Biopharm, Darmstadt, Germany), according to manufacturer’s instructions.

### 2.5. Microbial Community Composition (Test 3)

After 48 h of incubation, samples from both lumen and mucus were collected for analysis of the microbial community composition through quantitative polymerase chain reaction (qPCR) and 16S-targeted Illumina sequencing. DNA was isolated as described in [36] from either 1 mL luminal samples or 0.1 g mucus samples. Subsequently, qPCR was performed on a QuantStudio 5 Real-Time PCR system (Applied Biosystems, Foster City, CA, USA). Each sample was run in technical triplicate and outliers with more than 1 C_T_ difference were omitted. The qPCRs were performed as described previously for the following groups: *Lactobacillus* spp. [37], *Bifidobacterium* spp. and *Eubacterium rectale/Clostridium coccoides* [38], *Akkermansia muciniphila* [39], *Bacteroidetes* [40], *Enterobacteriaceae* [41], *Faecalibacterium prausnitzii* [42], *Roseburia* and *Eubacterium hallii* [43]. In addition, microbiota profiling was performed using 16S-targeted Illumina sequencing analysis (LGC genomics GmbH, Berlin, Germany) as described in [44] to obtain proportional abundances (%) at different phylogenetic levels (phylum, family, and operational taxonomic unit (OTU) level). Briefly, library preparation and sequencing were performed on an Illumina MiSeq platform with v3 chemistry. The 16S rRNA gene V3-V4 hypervariable regions were amplified using primers 341F (5′-CCT ACG GGN GGC WGC AG-3′) and 785Rmod (5′-GAC TAC HVG GGT ATC TAA KCC-3′) [45]. As described in [46,47], the 16S-targeted sequencing analysis was adapted from the MiSeq protocol for read assembly and cleanup using the mothur software (v. 1.39.5) as follows: (1) reads were assembled into contigs, (2) alignment-based quality filtering was performed by alignment to the mothur-reconstructed SILVA SEED alignment (v. 123), (3) chimeras were removed, (4) taxonomy was assigned via a naïve Bayesian classifier [48] and RDP release 14 [49] and (5) contigs were clustered into OTUs at 97% sequence similarity. Sequences classified as Eukaryota, Archaea, Chloroplasts, Mitochondria, and non-classified sequences were also removed. For each OTU, representative sequences were selected as the most abundant sequence within that OTU. Finally, the obtained high-resolution proportional phylogenetic information (i.e., proportional abundances (%)) was combined with an accurate quantification of total bacterial cells via flowcytometry to obtain quantitative data at phylum, family, and OTU level. This was done by multiplying the proportional abundances with absolute cell numbers (cells/mL) obtained via flowcytometry. For flowcytometry analysis, 10-fold serial dilutions were prepared in Dulbecco’s Phosphate-buffered Saline (DPBS) (Sigma–Aldrich, Bornem, Belgium) of all samples and stained with 0.01 mM SYTO24 (Life Technologies Europe, Merelbeke, Belgium) for 15′ at 37 °C in the dark. Samples were analyzed on a BD Facsverse (BDBiosciences, Erembodegem, Belgium) using the high-flow-rate setting and bacteria were separated from medium debris and signal noise by applying a threshold level of 200 on the SYTO channel. Flowcytometry data were analyzed using FlowJo, version 10.5.2.

### 2.6. Caco-2/PBMC Co-Culture Model (Test 4)

Caco-2 cells (HTB-37; American Type Culture Collection) were cultured in Dulbecco’s Modified Eagle Medium (DMEM) containing glucose and glutamine (Sigma–Aldrich, Bornem, Belgium) and supplemented with HEPES (4-(2-hydroxyethyl)-1-piperazineethanesulfonic acid) and 20% (v/v) heat-inactivated (HI) fetal bovine serum (FBS) (Gibco, Life Technologies Europe, Merelbeke, Belgium). PBMCs were isolated from buffy coats of healthy donors (Red Cross, Ghent, Belgium) using Lymphoprep^TM^ (STEMCELL technologies SARL, Grenoble, France). In brief, blood was collected and diluted (1/5, v/v) in DPBS without Ca/Mg (Sigma–Aldrich, Bornem, Belgium). Then, Lymphoprep^TM^ solution was added and samples were centrifuged at 1027× *g* for 20′ at room temperature (RT) to separate the mononuclear cells (MNCs) and red blood cells by density-gradient centrifugation. MNCs were collected and washed 3 times with ice-cold DPBS (340× *g*, 7′). Aliquots of PBMCs were frozen in liquid nitrogen. Baseline IL-8 levels were determined by enzyme-linked immunosorbent assay (ELISA) (Invitrogen, Thermo Fisher Scientific, Merelbeke, Belgium) to eliminate donors with high basal cytokine levels. For experiments, Caco-2 cells were seeded on 24-well semipermeable inserts (0.4 μm pore size) at a density of 1 × 10^5^ cells/insert and cultured for 14 days until a functional cell monolayer with a transepithelial electrical resistance (TEER) of more than 300 Ωcm^2^ was obtained. PBMCs, stimulated with 2.5 µg/mL pokeweed mitogen (PWM) were added to the basolateral chamber at a concentration of 1 *×* 10^6^ cells/well. PBMCs without PWM stimulation were included as negative control. At the same time, colonic suspensions (filter-sterilized (0.22 µm) and diluted 1/5 (v/v) in culture medium) or 5 mM sodium butyrate (NaB) (Sigma–Aldrich, Bornem, Belgium) were added to the Caco-2 cells at the apical side. Caco-2 cells were also treated with medium as negative control. Cells were incubated for 48 h at 37 °C in a humidified atmosphere of air/CO_2_ (95:5, v/v). TEER was measured at start (0 h timepoint) and after 48 h of incubation using a Millicell ERS2 Voltohmmeter (EMD Millipore, Sigma–Aldrich, Bornem, Belgium). All 48 h values were normalized to their own 0 h value after subtraction of the empty insert value and are presented as percentage of initial value. In addition, basolateral supernatant was collected after 48 h of incubation for cytokine analysis. Human IFN-γ, IL-17A, IL-21, IL-22, IL-4, and IL-9 levels were determined by Luminex^®^ multiplex (Procartaplex, Invitrogen, Thermo Fisher Scientific) and IL-10 levels were measured by ELISA (Invitrogen, Thermo Fisher Scientific), according to the manufacturers’ instructions. All experiments were performed in triplicate.

### 2.7. Statistics

To evaluate differences in microbial metabolites (tests 2 and 3) and microbial community composition at phylum level between blank and treatment incubations (Test 3), a two-way analysis of variance (ANOVA) with Dunnett’s multiple comparisons test was performed. Statistically significant differences between the blank and treatments are presented by (*, ∆0 h–6 h), ($, ∆6 h–24 h) or (#, ∆24 h–48 h). 1 sign = *p* < 0.05, 2 signs = *p* < 0.01, 3 signs = *p* < 0.001 and 4 signs = *p* < 0.0001. Statistical analysis was performed with the GraphPad Prism software (version 8.3.0, San Diego, USA). To evaluate differences in microbial community composition at family and OTU level between blank and treatment incubations (Test 3), a Student’s t-test was performed (Excel Software). Differences were found significant if *p* < 0.05. To evaluate differences between PWM+ and PWM- or NaB in the Caco-2/PBMC co-culture assay (Test 4), an ordinary one-way ANOVA with Dunnett’s multiple comparisons test was performed; while differences between blank and treatment incubations were assessed with an ordinary one-way ANOVA with Tukey’s multiple comparisons test. Statistically significant differences are presented by (*). (*) = *p* < 0.05, (**) = *p* < 0.01, (***) = *p* < 0.001 and (****) = *p* < 0.0001. Statistical analysis was performed with the GraphPad Prism software (version 8.3.0, San Diego, USA).

## 3. Results

### 3.1. cRG-I is Resistant to Digestion in the Human Upper Gastro-Intestinal Tract (GIT) (Test 1)

**Figure 2:**
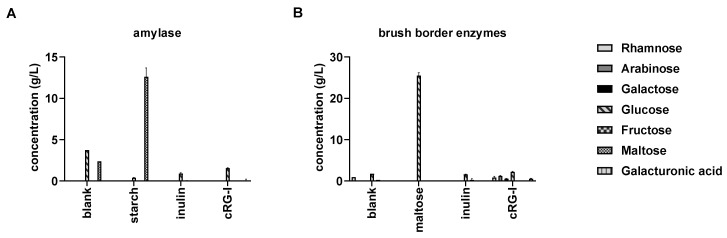
Digestion of cRG-I (carrot-derived rhamnogalacturonan-I) by amylase (**A**) and brush border enzymes (**B**) versus inulin, cooked starch, and maltose. Blank incubations containing all reagents in absence of a substrate were included. Average (± st. dev.) concentrations of different monosaccharides and maltose were measured by High Performance Anion Exchange Chromatography (HPAEC) (*n* = 3).

One of the characteristics of a prebiotic is its non-digestibility by host enzymes upon passage along the upper GIT [19]. First, upon exposure to amylase, the positive control cooked starch was readily degraded into maltose, demonstrating its known digestibility (Figure 2A), while the negative control inulin and also cRG-I were not digested to any of the simple sugars measured. Likewise, maltose was digested by brush border enzymes into glucose (Figure 2B), in contrast to the negative control inulin and cRG-I. Therefore, cRG-I can be considered to be a polysaccharide that likely escapes upper GIT digestion in vivo thereby reaching the colon where it could be fermented by the gut microbiota.

### 3.2. Effect of cRG-I on Microbial Metabolic Activity in Short-Term Colonic Incubations (Test 2)

**Figure 3:**
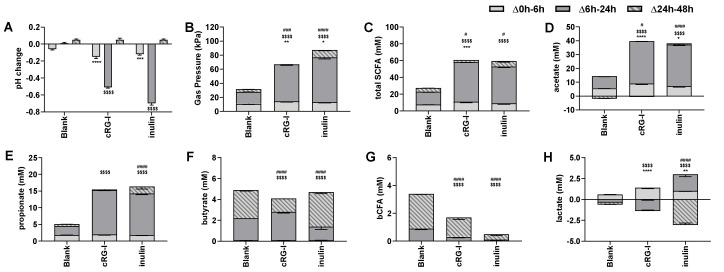
Effect of fermentation of cRG-I on microbial metabolic activity in short-term colonic incubations. Average changes (± st. dev.) in pH (**A**), gas production (**B**), total short-chain fatty acids (SCFA) (**C**), acetate (**D**), propionate (**E**), butyrate (**F**), branched CFA (bCFA) (**G**) and lactate (**H**) levels between 0 and 6 h (light gray), 6 h and 24 h (dark gray) and 24 h and 48 h (stripes) upon dosing cRG-I and inulin to the gut microbiota versus a blank incubation (*n* = 3). Statistically significant differences between blank and treatments for different time intervals are presented by * for Δ0 h–6 h, $ for Δ6 h–24 h or # for Δ24 h–48 h. (*, $, #) = *p* < 0.05, (**, $$, ##) = *p* < 0.01, (***, $$$, ###) = *p* < 0.001 and (****, $$$$, ####) = *p* < 0.0001.

Short-term colonic incubations were performed to investigate the potential prebiotic effect of cRG-I on microbial activity, including inulin as a positive control. A first indication of cRG-I fermentation by the gut microbiota followed from the significant pH decrease and enhanced gas production during the first 6 h of incubation (Δ0–6 h) versus the blank (Figure 3A,B). These changes were even stronger compared to inulin. Between 6 h and 24 h, cRG-I significantly and strongly decreased pH while increasing gas production. pH decreases and gas production were more excessive for inulin. Finally, during the 24 h to 48 h time interval, relatively stable pH values and gas levels indicated substrate depletion.

Upon measuring lactate and SCFA production, acids responsible for aforementioned pH changes were elucidated. During the 0 h to 6 h time interval, cRG-I, but not inulin, significantly increased the production of total SCFA (Figure 3C) which resulted from more strongly elevated acetate levels (Figure 3D) next to significant raises in lactate production for cRG-I (Figure 3H). Furthermore, both treatments strongly augmented total SCFA production during the 6 h to 24 h time interval (merely due to increases in acetate and propionate (Figure 3D,E)), which were more profound for cRG-I compared to inulin. cRG-I, unlike inulin, also stimulated butyrate production during this interval (Figure 3F), which coincided with lactate consumption. Finally, during the 24 h to 48 h time interval, total SCFA production only increased upon treatment with inulin, which was related to increases of acetate, propionate, and butyrate levels, thus indicating slower fermentation of inulin versus cRG-I (Figure 3C–F). The stimulation of butyrate by inulin between 24–48 h correlated with the consumption of lactate during this time interval. Overall, lactate was entirely consumed at the end of the incubations, suggesting optimal conversion to propionate and/or butyrate [50]. Finally, bCFA result from protein fermentation by the gut microbiota [51,52], which is associated with detrimental health effects [51]. bCFA were mainly produced during the 6 h to 48 h time frame (Figure 3G), with both cRG-I and especially inulin significantly decreasing bCFA production.

### 3.3. Effect of cRG-I and cRG-I +LMWC on Microbial Metabolic Activity in Short-Term Colonic Incubations with or without a Mucosal Compartment (Test 3)

**Figure 4:**
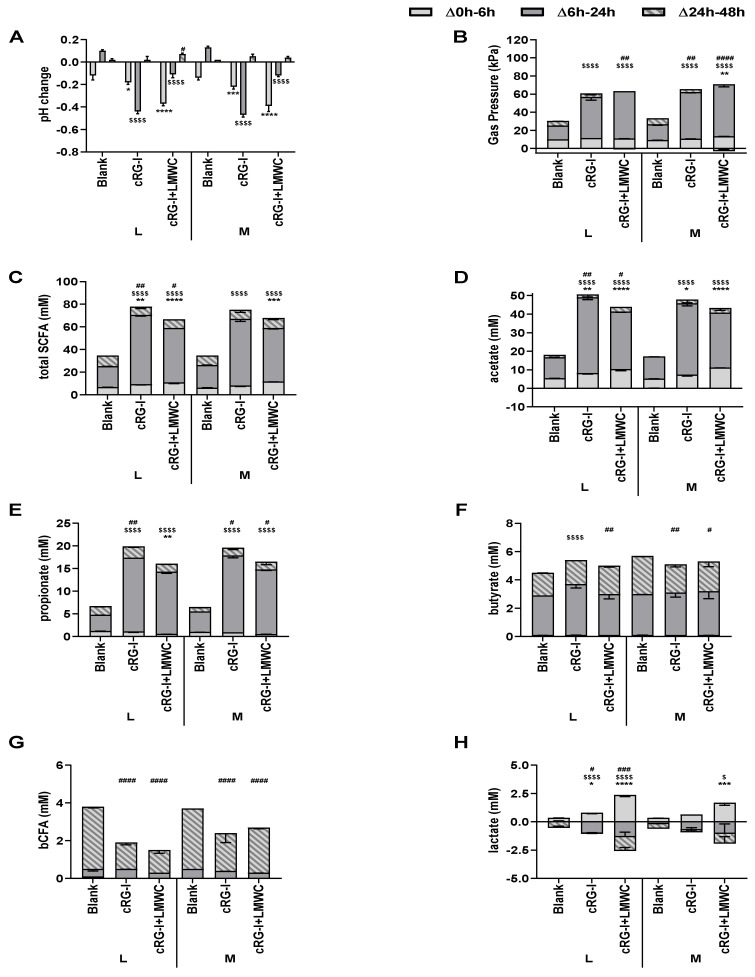
Effect of fermentation of cRG-I and cRG-I+LMWC on microbial metabolic activity in short-term colonic batch simulations in absence or presence of a mucosal compartment. Average changes (± st. dev.) in pH (**A**), gas production (**B**), total short-chain fatty acids (SCFA) (**C**), acetate (**D**), propionate (**E**), butyrate (**F**), branched CFA (bCFA) (**G**) and lactate (**H**) levels between 0 and 6 h (light gray), 6 h and 24 h (dark gray) and 24 h and 48 h (stripes) upon dosing cRG-I and cRG-I + LMWC to the gut microbiota versus a blank incubation (*n* = 3). Statistically significant differences between the blank and treatments for different time intervals are presented by * for Δ0 h–6 h, $ for Δ6 h–24 h or # for Δ24 h–48 h). (*, $, #) = *p* < 0.05, (**, $$, ##) = *p* < 0.01, (***, $$$, ###) = *p* < 0.001 and (****, $$$$, ####) = *p* < 0.0001. L = colonic incubations only simulating lumen; M = incubations simulating both lumen and mucus.

An in-depth characterization of the effects of cRG-I on microbial metabolic activity (Figure 4A–H) was performed by testing both cRG-I and a modified formulation containing small sugars (cRG-I +LMWC), dosed to colonic incubations including solely a luminal (L) or additionally also a mucosal (M) environment. In consistency with Test 2, cRG-I significantly decreased the pH during the first 6 h of incubation versus the blank due to enhanced acetate and lactate levels. Furthermore, the main fermentation occurred between 6 h and 24 h with strong increases in gas production and further decreases of pH due to stimulation of acetate, propionate, and to a lesser extent butyrate. The latter could again be linked to coinciding lactate consumption. Finally, the absence of marked changes of aforementioned parameters between 24 h and 48 h again indicated substrate depletion, while bCFA were significantly decreased thus illustrating the potential protective effects of cRG-I against toxic by-products of proteolytic fermentation.

cRG-I+LMWC exerted similar effects on microbial activity versus cRG-I with some minor differences. These included a more profound pH decrease for cRG-I+LMWC during the first 6 h of incubation due to enhanced acetate and lactate levels. Furthermore, acetate and propionate production between 6–24 h was less strongly increased with cRG-I+LMWC compared to cRG-I.

Overall, while no major differences were observed between incubations with or without mucosal environment, inclusion of mucus beads led to a tendency to higher gas production, with higher butyrate levels for the blank incubation, suggesting colonization of butyrate-producing bacteria on the mucin-coated carriers.

### 3.4. Effect of cRG-I and cRG-I +LMWC on Microbial Community Composition in Short-Term Colonic Incubations with or without a Mucosal Compartment (Test 3)

**Figure 5:**
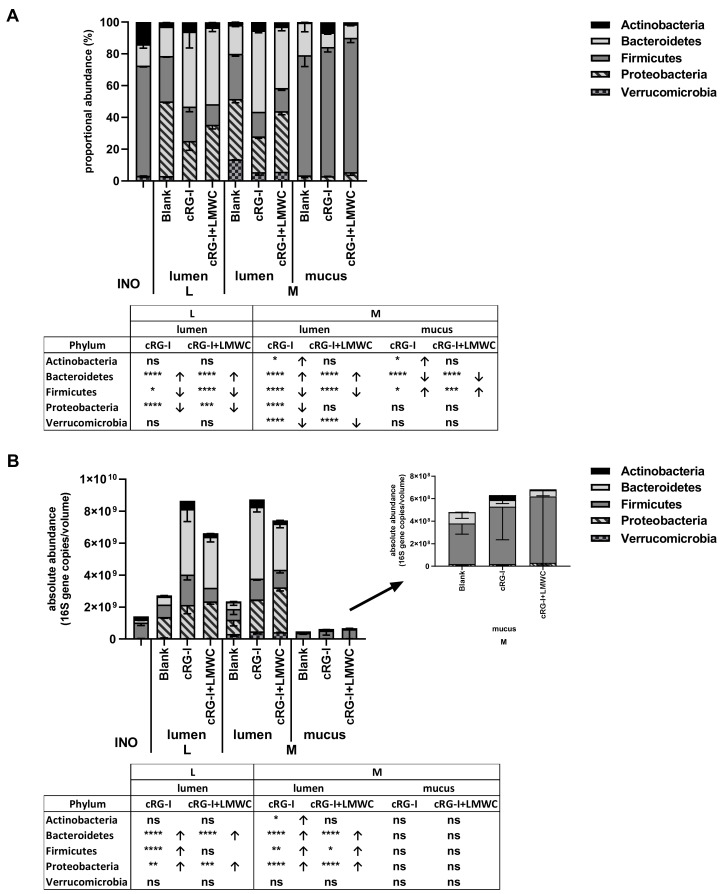
Effect of fermentation of cRG-I and cRG-I+LMWC on microbial community composition at phylum level in short-term colonic incubations in absence or presence of a mucosal compartment. Average (± st. dev.) proportional (%) (**A**) and absolute (16S gene copies/mL) (**B**) abundance of the different phyla in the original (diluted) inoculum (INO) and after 48 h of incubation upon dosing of cRG-I and cRG-I+LMWC versus a blank control (n = 3). Statistically significant differences between the blank and treatments are presented by *. (*) = *p* < 0.05, (**) = *p* < 0.01, (***) = *p* < 0.001 and (****) = *p* < 0.0001. L = colonic incubations only simulating lumen; M = incubations simulating both lumen and mucus.

The data at phylum level are presented both as proportional (Figure 5A) and absolute values (Figure 5B). This demonstrated that for the luminal microbiota, quantitative data revealed greater insight in the true compositional changes since both cRG-I and cRG-I+LMWC largely increased total cell numbers. In contrast, due to the large variation in total cell numbers within identical replicates for the mucosal microbiota resulting in large variations of quantitative numbers, proportional abundances were preferred to draw conclusions on modulation of the mucosal microbiota. Therefore, abundances at the family level are presented as absolute data for the luminal microbiota, whereas they are presented as proportional values for the mucosal compartment (Table 1). Furthermore, proportional abundance of the 25 most abundant OTUs and 7 additional OTUs affected by at least one of the treatments are shown in Supplementary Table S1 to get insights at the highest phylogenetic resolution possible.

First, upon comparing the luminal and mucosal microbiota, it followed that the Firmicutes phylum was enriched in the mucosal compartment, while *Actinobacteria*, *Proteobacteria* and *Verrucomicrobia* were enriched in the lumen (Figure 5A), in accordance to what has been published for the M-SHIME^®^ model [12]. At family level, the mucosal *Firmicutes* enrichment was due to a marked enrichment in *Clostridiaceae* cluster I and *Lachnospiraceae* (Table 1). At OTU level, this was reflected by an enrichment of OTU14 (related to *Clostridium butyricum*), OTU10 (related to *Clostridium tertium*), OTU8 (related to *Clostridium paraputrificum*), OTU12 (related to *Ruminococcus torques*), OTU3 (related to *Roseburia hominis*) and OTU19 (related to *Ruminococcus lactaris*) (Table S1). Furthermore, the decreased mucosal levels of *Actinobacteria*, *Proteobacteria* and *Verrucomicrobia* were solely related to a decreased mucosal colonization of members of the *Eggerthellaceae*, *Enterobacteriaceae*, and *Akkermansiaceae*. Another overall finding of the in vitro model was that the luminal microbiota of blank incubations in absence and presence of a mucosal environment were highly similar. The introduction of mucin beads only resulted in an enrichment of the luminal *Verrucomicrobia* levels (Figure 5A,B). At family level, this was due to an increased luminal abundance of *Akkermansiaceae* (+0.55 log copies/mL) (Table 1).

With respect to treatment effects in the lumen, cRG-I and cRG-I+LMWC both increased the absolute levels of *Actinobacteria*, *Bacteroidetes* and *Proteobacteria* compared to the blank (Figure 5B). Although the increase of *Actinobacteria* and *Bacteroidetes* was strongest for cRG-I, the increase in *Proteobacteria* was strongest for cRG-I+LMWC. cRG-I additionally increased luminal *Firmicutes* levels. The luminal increase in *Actinobacteria* by cRG-I was due to a significant increase of OTUs related to *Bifidobacterium longum* (OTU7) and *Bifidobacterium adolescentis* (OTU21) (Table S1), thus also strongly increasing *Bifidobacteriaceae* levels upon cRG-I treatment (Table 1). Furthermore, cRG-I also stimulated mucosal *Bifidobacteriaceae*, mostly due to the stimulation of the *Bifidobacterium longum*-related OTU7. The luminal *Bacteroidetes* increase upon treatment with both products was due to the stimulation of the *Bacteroidaceae* and *Prevotellaceae* families, with again the highest levels being reached for cRG-I. At OTU level, a wide spectrum of *Bacteroidaceae* members were stimulated upon dosing both cRG-I and cRG-I+LMWC, including OTUs related to *B. ovatus* (OTU22), *B. plebeius* (OTU6), *B. xylanisolvens* (OTU18) and especially *B. dorei* (OTU2). As a remark, a decrease in abundances of OTUs related to *B. caccae* (OTU13), *B. fragilis* (OTU11) and *B. uniformis* (OTU17) upon cRG-I and cRG-I+LMWC treatment was noted, but these decreases were less profound compared to observed increases in other OTUs. The increased abundance in *Proteobacteria* related to an enrichment of *Desulfovibrionaceae* and *Enterobacteriaceae*, which was most pronounced upon dosing of cRG-I+LMWC. Finally, the luminal increase in *Firmicutes* with cRG-I and cRG-I+LMWC was due to increased levels of *Erysipelotrichaceae*, *Peptostreptococcaceae*, *Streptococcacae*, *Ruminococcaceae*, and *Veillonellaceae*. The enrichment in *Streptococcacae* was most pronounced upon dosing cRG-I+LMWC and linked to an increase in OTU26 (related to *Streptococcus aginosus*). In contrast, the increase in *Veillonellaceae* was more pronounced upon dosing of cRG-I and was attributed to an increase in OTU9 (related to *Dialister succinatiphilus*). Within the *Ruminococcaceae*, two OTUs related to *Faecalibacterium prausnitzii* (i.e., OTU83 and OTU5) increased upon cRG-I, while only OTU5 was stimulated by cRG-I+LMWC and this to a lesser extent.

In the mucosal compartment, both treatments slightly increased *Firmicutes*, while decreasing *Bacteroidetes* levels (Figure 5A). Both cRG-I and cRG-I+LMWC strongly enriched *Lachnospiraceae* (Table 1), due to a marked stimulation of OTU3 (related to *Roseburia hominis*) that increased from 9.8% in the blank control incubations to 64.3% and 74.2%, respectively (Table S1). Although both treatments also significantly increased the proportion of *Streptococcacae* in the mucosal environment, only cRG-I increased mucosal *Actinobacteria* abundances.

To confirm several of the above-mentioned observations obtained through 16S-targeted Illumina sequencing, qPCRs on specific bacterial groups of interest were performed (Supplementary Table S2). This confirmed the key aforementioned conclusions obtained through 16S-targeted Illumina sequencing including that both cRG-I and cRG-I+LMWC stimulated levels of (i) luminal/mucosal *Bifidobacteriaceae* (and not *Lactobacillaceae*); (ii) luminal *Bacteroidetes*; (iii) luminal *Faecalibacterium prausnitzii*; (iv) mucosal *Roseburia* (and *Eubacterium rectale/Clostridium coccoides* group to which it belongs); while not affecting *Akkermansia muciniphila*. In addition, both products, but mostly cRG-I+LMWC, increased colonization of *Eubacterium hallii* in the simulated mucus layer.

### 3.5. Effect of Fermented cRG-I on Intestinal Epithelial Barrier (Test 4)

**Figure 6:**
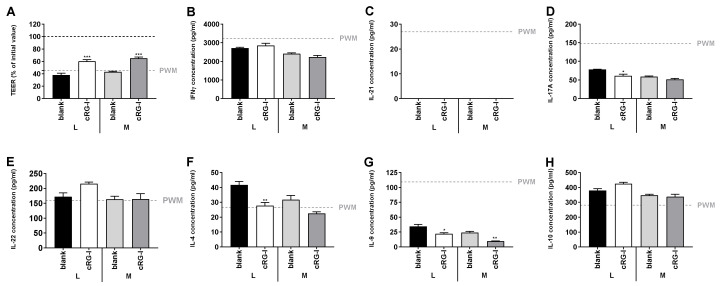
Effect of fermented cRG-I on transepithelial electrical resistance (TEER) and cytokine production in a Caco-2/PBMC co-culture system. Caco-2 cells, cultured 14 days on transwell inserts, were placed on top of pokeweed mitogen (PWM)-activated PBMCs and incubated for 48 h at the apical side with blank or treatment samples collected during the colonic batch incubations containing only a luminal (L) or also a mucosal (M) environment. Average (±SEM) TEER of the Caco-2 monolayers (**A**) and concentration of secreted interferon (IFN)γ (**B**), interleukin (IL)-17A (**C**), IL-21 (**D**), IL-22 (**E**), IL-4 (**F**), IL-9 (**G**) and IL-10 (**H**) in the basolateral medium are shown (n = 3). Statistically significant differences to PWM are represented by (*). (*) = *p* < 0.05, (**) = *p* < 0.01, (***) = *p* < 0.001.

As cRG-I fermentation enriched several health-associated species and increased production of SCFA, it might exert favorable effects at the host level. To address this question, a Caco-2/PBMC co-culture model was developed. Inflammation-induced barrier disruption was obtained upon 48 h co-culturing of Caco-2 cells with PWM-activated PBMCs and measured as a significant decrease in TEER of the Caco-2 monolayers (Figure S1A). In addition, apical treatment with the positive control sodium butyrate (NaB) prevented this TEER decrease. Likewise, apical treatment with fermented cRG-I showed a significant increase in TEER compared to the blank controls (Figure 6A). Furthermore, effects on T-cell dependent cytokine production were assessed. As shown in Figure S1, NaB significantly decreased the secretion of interferon (IFN)γ, interleukin (IL)-17A, IL-21, IL-4, and IL-9; while increasing the secretion of IL-22; a cytokine involved in maintenance of barrier integrity, wound healing and antimicrobial responses [53]. However, in contrast to its positive effects on IL-10 secretion in the Caco-2/THP1 co-culture assay [29], NaB significantly decreased IL-10 secretion possibly due to toxicity of NaB on IL-10 producing cells at the concentration used [54,55]. Compared to the blank control, fermented cRG-I reduced the secretion of IL-17A, IL-4, and IL-9; reaching statistical significance for IL-17A and IL-4 upon luminal incubations and upon both incubations for IL-9 (Figure 6D,F,G). Furthermore, luminal fermentation of cRG-I tended to increase the secretion of IL-22 and IL-10; of which the latter was already increased upon treatment with the blank controls (Figure 6E,H). In addition, all colonic suspensions completely abolished PWM-induced IL-21 secretion (Figure 6C); while no significant differences were observed on IFNγ secretion (Figure 6B). Hence, metabolites generated from colonic fermentation of cRG-I displayed anti-inflammatory and gut barrier protective properties.

Overall, some minor effects of the incorporation of a mucosal compartment were observed as both blank and treatment colonic suspensions slightly increased TEER; while decreased the secretion of all tested cytokines compared to luminal suspensions (Figure 6).

## 4. Discussion

In the current study, novel in vitro models and analytical techniques were implemented to investigate whether cRG-I classifies as a potential prebiotic ingredient. First, upon exposure to α-amylase and brush border enzymes (Test 1), unlike the positive controls (cooked starch and maltose, respectively), cRG-I (like the negative control inulin) was not digested to any of the simple sugars measured. This suggests that cRG-I can be considered to be a polysaccharide that likely escapes upper GIT digestion in vivo, thus fulfilling the definition of a prebiotic ingredient that should reach the colon where it could be fermented by the gut microbiota. Secondly, a recently described short-term colonic incubation strategy [29] was upgraded with a simulation of the mucosal microbiota (Test 3). By applying a novel technique to analyze the microbial community composition, i.e., quantitative 16S-targeted Illumina sequencing [33], in-depth quantitative information was obtained at high phylogenetic resolution. Besides elucidating treatment effects of cRG-I, this allowed to validate the implementation of mucin-coated carriers in the short-term incubations by demonstrating a relevant species-specific colonization of the mucosal environment. Indeed, consistent with the well-established long-term M-SHIME^®^ model [12], a wide spectrum of (potential butyrate-producing) *Firmicutes* members specifically colonized the mucosal environment (e.g., OTUs related to *Clostridium butyricum* and *Roseburia hominis*). Furthermore, inclusion of the mucosal environment did not alter the luminal microbiota (except for a minor enrichment in *Akkermansiaceae*), nor did it alter the treatment effects towards luminal microbiota activity and community composition. Overall, including the mucin beads in the current colonic simulations allowed to maintain a higher diversity, thus allowing observation of more complete treatment effects of cRG-I. Finally, application of a model combining gut epithelial and immune cells allowed to point out the gut protective effect of cRG-I fermentation-derived metabolites against an inflammatory stressor (Test 4).

In a first series of short-term colonic incubations (Test 2), cRG-I was found to display prebiotic potential comparable but not identical to that of inulin in modulating microbial activity [19], as followed from increases in health-promoting SCFA (acetate and propionate) and lactate and decreases of bCFA. A side effect of inulin fermentation is the production of high amounts of gas that has been observed in in vitro experiments and clinical studies. Depending on the dose, this can result in mild negative gastro-intestinal symptoms [56,57,58]. Interestingly, gas production upon dosing of cRG-I was milder compared to inulin, which suggests that in vivo consumption of cRG-I could be less accompanied by adverse side-effects such as bloating and abdominal pain.

Upon fermentation in both short-term colonic incubations (Tests 2/3), cRG-I stimulated acetate, and lactate production which was accompanied with a strong reduction in pH. At community level, this correlated with increases in OTUs related to *Bifidobacterium longum* (OTU7) and *Bifidobacterium adolescentis* (OTU21). *Bifidobacteriaceae* are indeed key acetate and lactate producers [59,60]. Although health effects are to be considered strain-specific, multiple *Bifidobacterium* strains have been associated with beneficial effects on the host health and some strains are widely used as probiotics. A first mechanism by which *Bifidobacteriaceae* contribute to health is by indirectly promoting butyrate production via cross-feeding mechanisms involving for instance *Faecalibacterium prausnitzii* and *Eubacterium hallii* [59,61,62], taxa of which related OTUs were indeed found to be increased upon cRG-I treatment in the current study. In addition, acetate produced by bifidobacteria was also shown to play a key role in their anti-infectious properties against enteropathogens [63]. In terms of host effects, a specific *B. longum* strain has e.g., been shown to exert protective effects in a DSS-induced colitis model in mice by reducing inflammation and enhancing the intestinal epithelial barrier [64]. Moreover, a specific *B. longum* strain reduced chronic mucosal inflammation in ulcerative colitis (UC) patients in a double-blinded, randomized-controlled clinical trial [65]. Likewise, a specific *B. adolescentis* strain protected mice from DSS-induced colitis by increasing IL-10 levels, up-regulation of regulatory T-cells (T_reg_) and decreasing IL-17A positive T-cells [66]. The strong bifidogenic effect exerted by cRG-I thus supports the prebiotic potential of this novel food ingredient.

Next to acetate and lactate, fermentation of cRG-I increased the production of propionate, which correlated with a stimulation of *Prevotellaceae* and *Bacteroidaceae* due to the stimulation of a wide spectrum of *Bacteroidaceae* OTUs related to e.g., *B. ovatus*, *B. plebeius*, *B. xylanisolvens* and especially *B. dorei*. Indeed, *Bacteriodetes* spp. are known primary fiber degraders that are capable of producing propionate [50]. In the colon, health-related effects of propionate are related to anti-cancer effects [7,8]. Furthermore, propionate was shown to exert anti-lipogenic and cholesterol-lowering effects in the liver [6]. Finally, propionate is a satiety-inducing agent affecting energy intake and feeding behavior [67]. Besides the beneficial effects of propionate, the aforementioned stimulations of specific *Bacteroidaceae* members have also been related to particular health benefits. As an example, in a mouse model of atherosclerosis, *B. dorei* reduced plaque inflammation and decreased intestinal epithelial permeability and systemic endotoxemia [68]. Furthermore, *B. ovatus* reduced mucosal inflammation and stimulated epithelial proliferation and mucin production in a DSS-induced colitis model in mice [69]. Another microbial modulation that could have boosted propionate production upon cRG-I treatment was the increase in *Veillonellaceae* that was solely attributed to an increase in OTU9 (related to *Dialister succinatiphilus*). Interestingly, *D. succinatiphilus* is a succinate-converting, propionate-producing species [70] and as many *Bacteroidetes* spp. are known succinate producers [50], its increase might have contributed to the stronger increase in propionate levels upon dosing of cRG-I. As a final remark, not all *Bacteroidaceae* members increased upon cRG-I treatment, with OTUs relating to *B. caccae* and *B. fragilis* decreasing in abundance upon cRG-I treatment. These species are considered to be opportunistic pathogens carrying virulence factors: enterotoxigenic *B. fragilis* strains secrete the *B. fragilis* toxin (BFT) [71] and *B. caccae* contains the *ompW* gene [72]. Besides its strong bifidogenic effect, cRG-I could further exert health benefits by targeted modulation of specific propionate-producing taxa.

In contrast to propionate, butyrate was not significantly increased upon dosing of cRG-I. However, cRG-I did strongly stimulate *Lachnospiraceae*, containing several butyrate-producing species, in the mucosal compartment. The increase in *Lachnospiraceae* was related to a strong stimulation of an OTU related to *Roseburia hominis*, which has been associated with beneficial effects on barrier function and immune regulation in the gut [73]. Indeed, *R. hominis* showed protective effects in a DSS-model for colitis in mice by reducing pro-inflammatory cytokine expression. Moreover, increased T_reg_ levels were observed in both germ-free and conventional mice fed a supplement containing live *R. hominis*. Also, a significant reduction of *R. hominis* and *F. prausnitzii* was observed in UC and Crohn’s disease (CD) patients [74,75]. Interestingly, cRG-I increased the abundance of OTUs related to butyrate-producing species such as *F. prausnitzii* and *Eubacterium hallii*. Like *R. hominis*, *F. prausnitzii* was shown to exert anti-inflammatory potential by increasing IL-10 and promoting T_reg_ differentiation in mice [76]. On the other hand, *E. hallii* improved insulin sensitivity in a mouse model for diabetes [77]. Of note, discrepancy between the stimulation of butyrate producers in the mucosal environment with cRG-I versus the absence of treatment effects on butyrate levels might be explained by the fact that the biofilm which develops on the mucin-coated carriers during short-term colonic incubations (48 h) is still developing during cRG-I treatment. Hence, by the time the biofilm is developed (48 h), all substrate has been consumed (in fact already after 24 h). This may potentially limit the detection of treatment effects resulting from modulation of mucosal microbes on metabolic activity (particularly butyrate production) during short-term incubations. Therefore, testing the impact of cRG-I in a long-term M-SHIME^®^ study could further elucidate the potential impact of cRG-I on butyrate production.

The stimulation of several health-related microbial species and the concomitant increase in health-promoting metabolites suggested that cRG-I may display interesting host beneficial properties in terms of intestinal barrier protection and reduction of inflammation. This hypothesis was confirmed using a Caco-2/PBMC co-culture model in which treatment with fermented cRG-I significantly increased the TEER, indicative for the protective effects of fermentation-derived cRG-I metabolites on inflammation-induced intestinal permeability. To further strengthen this observation, it would be interesting to perform in-depth analysis of the expression and localization of tight junction proteins including occludin, ZO-1, and claudins upon cRG-I treatment in this system. Furthermore, fermented cRG-I metabolites decreased the secretion of the pro-inflammatory cytokines IL-17A, IL-4, and IL-9, while increasing the secretion of IL-22 and of the anti-inflammatory IL-10. This is suggestive of a possible immunoregulatory effect of cRG-I metabolites on the T_reg_/T_H_17 axis; favoring down-regulation of excessive inflammation as IL-10 is necessary for T_reg_ functions [78]. Th17 cells have a dual role in human health as although required for clearance of extracellular pathogens, an elevated frequency of Th17 cells associated with impaired T_reg_ functions has been reported in IBD and other extraintestinal autoimmune disorders [79]. Furthermore, IL-22 plays a role in maintaining the integrity of the mucosal barrier by promoting wound healing and activating antimicrobial responses [53]. Finally, increased levels of IL-4 and IL-9 were associated with UC [80,81]. In addition, IL-9 inhibits wound healing in the intestinal mucosa and impairs intestinal epithelial barrier functions. Also, IL-9 directly regulates tissue recruitment and inflammatory functions of mast cells. Together, these data suggest an interesting immuno-modulatory role of cRG-I metabolites in the gut in terms of increasing barrier tightness and prevention of a “leaky gut”.

Finally, a modified formulation of cRG-I was tested which contained small size sugars, i.e., cRG-I+LMWC. Interestingly, the short-term colonic incubation strategy applied in this study was highly sensitive to pick up differences in closely related product compositions. For instance, the presence of these simple sugars resulted in a stronger initial pH decrease upon dosing of cRG-I+LMWC, related to an initial increased production of lactate, which could be linked to a stronger enrichment in a *Streptococcacae* OTU related to *Streptococcus aginosus*. Also, *Enterobacteriaceae* levels were higher upon cRG-I+LMWC treatment. Like *Streptococcaceae*, *Enterobacteriaceae* are expert fermenters of simple sugars [82], specifically present at higher levels in this preparation. Finally, *Coriobacteriaceae* (OTU15 related to *Collinsella aerofaciens*) and *Desulfovibrionaceae* tended to be higher in incubations with cRG-I+LMWC. This suggested a potential co-existence of e.g., *C. aerofaciens* and *Desulfovibrio piger*, based on the fact that the main products of *C. aerofaciens* fermentation (i.e., lactate, H_2_, and formate) serve as substrates for *D. piger*, which is the main sulfate-reducing bacterial species in the human gut microbiome [83]. This might also indicate that cRG-I+LMWC might contain small quantities of sulfur (sulfate or sulfur-containing amino acids). These data altogether stress the relevance of testing prebiotic candidates in short-term colonic in vitro incubations to understand their potential impact on the human gut microbiome and to support structure-function studies.

In conclusion, the implementation of novel in vitro models simulating the human colonic environment coupled to cell-based assays mimicking the host gut barrier, allowed to establish the prebiotic potential of cRG-I. This novel fiber is not digested by host enzymes characteristic of the upper gastro-intestinal tract but rapidly fermented by the human colonic microbiota leading to selective stimulation of the growth and activity of intestinal bacterial species associated with human health. cRG-I displays unique properties as it was fermented more rapidly than inulin leading to production of SCFA, especially acetate and propionate, and less gas than inulin. It increases the abundance of several bacterial species reputed for their “anti-inflammatory” profile. In line with this, the metabolites resulting from cRG-I fermentation exhibited a protective effect in an in vitro model of inflamed gut barrier. Overall, the data obtained during this study support future research to further investigate this novel prebiotic candidate in long-term SHIME^®^ models with different fecal sample donors and in clinical trials to confirm the beneficial effect of cRG-I on the microbiota and its impact on human health.

## Figures and Tables

**Table 1:** Effect of fermentation of cRG-I and cRG-I+LMWC on microbial composition at family level in short-term colonic incubations in absence or presence of a mucosal compartment. Average proportional (%) (mucus) and absolute (16S gene copies/mL) (lumen) abundance of the different bacterial families in the original (diluted) inoculum and after 48 h of incubation upon dosing cRG-I and cRG-I +LMWC versus a blank control (*n* = 3). Statistically significant differences between the blank and treatments are indicated in bold (*p* < 0.05). Upon reaching statistically significant differences, highest values are underlined. Values indicated in italicsy are strongly increased, although not significant. L = colonic incubations only simulating lumen; M = incubations simulating both lumen and mucus.

		Absolute Abundance (16S Gene Copies/mL)							Proportional Abundance (%)		
Phylum	Family	Inoculum	L			M					
			lumen			lumen			mucus		
			blank	cRG-I	cRG-I+LMWC	blank	cRG-I	cRG-I+LMWC	blank	cRG-I	cRG-I+LMWC
Actinobacteria	Bifidobacteriaceae	7.28	7.18	8.68	8.28	6.89	8.63	8.26	0.2%	6.4%	1.2%
	Coriobacteriaceae	8.22	6.60	6.39	6.88	6.25	6.36	6.80	0.0%	0.0%	0.0%
	Eggerthellaceae	6.56	7.72	**7.40**	**7.22**	7.49	7.29	7.09	0.1%	0.0%	0.0%
Bacteroidetes	Bacteroidaceae	7.94	8.63	**9.60**	**9.50**	8.52	**9.65**	**9.45**	20.1%	**9.1%**	**8.5%**
	Marinifilaceae	6.23	6.12	6.36	6.04	6.46	6.43	6.25	0.1%	0.0%	0.0%
	Muribaculaceae	6.10	7.02	6.73	**6.15**	6.78	6.56	6.03	0.1%	0.0%	**0.0%**
	Prevotellaceae	6.74	<LOQ	**7.04**	**6.04**	5.48	**6.55**	**6.15**	0.0%	0.0%	0.0%
	Rikenellaceae	7.94	6.85	7.02	6.60	6.57	6.83	6.68	0.1%	0.0%	0.1%
	Tannerellaceae	6.19	7.70	**7.35**	**7.05**	7.58	7.42	**7.24**	0.1%	0.0%	0.0%
Firmicutes	Christensenellaceae	7.58	7.06	7.14	**6.66**	6.82	6.94	6.75	0.0%	0.0%	0.0%
	Clostridiaceae cluster I	6.15	5.96	<LOQ	5.95	6.40	5.94	6.17	41.7%	**2.1%**	**1.8%**
	Erysipelotrichaceae	7.41	6.93	**7.32**	**7.33**	7.07	7.55	**7.81**	1.1%	1.0%	1.4%
	Clostridiaceae cluster XI	<LOQ	6.27	**6.58**	6.39	6.36	6.48	6.69	0.0%	0.0%	0.0%
	Clostridiaceae cluster XIII	6.43	6.45	6.14	<LOQ	6.73	6.16	**6.09**	0.0%	0.0%	0.0%
	Lachnospiraceae	8.59	8.65	**8.93**	8.57	8.51	8.73	8.58	28.3%	**74.0%**	**79.1%**
	Peptococcaceae	6.53	6.36	6.07	**5.85**	6.16	<LOQ	5.69	0.0%	0.0%	0.0%
	Peptostreptococcaceae	6.45	6.15	**6.70**	6.43	5.95	**7.16**	**7.30**	0.0%	0.0%	0.0%
	Ruminococcaceae	8.64	8.38	**8.83**	**8.30**	8.34	8.72	8.49	4.1%	3.0%	**1.5%**
	Streptococcaceae	6.37	<LOQ	**6.70**	**8.14**	5.26	5.80	**8.17**	0.1%	*1.0%*	**0.5%**
	Veillonellaceae	7.77	7.67	**8.45**	**8.01**	7.51	**8.31**	**8.16**	0.2%	0.2%	0.2%
Proteobacteria	Burkholderiaceae	5.18	6.78	6.83	**6.32**	6.74	6.60	6.41	0.0%	0.0%	0.0%
	Desulfovibrionaceae	6.19	6.26	**6.73**	**6.75**	6.15	**6.80**	**6.93**	0.0%	0.0%	0.0%
	Enterobacteriaceae	6.03	9.10	**9.32**	**9.35**	8.90	**9.29**	**9.45**	2.8%	2.8%	*5.1%*
Verrucomicrobia	Akkermansiaceae	7.63	7.91	7.55	7.71	8.46	8.67	8.64	0.6%	0.2%	0.4%

## Supplementary Materials

The following are available online at https://www.mdpi.com/2072-6643/12/7/1917/s1, Figure S1: Effect of Sodium butyrate (NaB) on transepithelial electrical resistance (TEER) and cytokine production in a Caco-2/PBMC co-culture system; Table S1: Effect of fermentation of cRG-I and cRG-I+LMWC on microbial community composition at OTU level in short-term colonic batch simulations in absence or presence of a mucosal compartment; Table S2: Effect of fermentation of cRG-I and cRG-I+LMWC on selected bacterial groups in short-term colonic batch simulations in absence or presence of a mucosal compartment as assessed through qPCR.

## References

[1] Forgie A.J., Fouhse J.M., Willing B.P. (2019). Diet-Microbe-Host Interactions That Affect Gut Mucosal Integrity and Infection Resistance. Front. Immunol..

[2] Monteagudo-Mera A., Rastall R.A., Gibson G.R., Charalampopoulos D., Chatzifragkou A. (2019). Adhesion mechanisms mediated by probiotics and prebiotics and their potential impact on human health. Appl. Microbiol. Biotechnol..

[3] Kachrimanidou M., Tsintarakis E. (2020). Insights into the Role of Human Gut Microbiota in Clostridioides difficile Infection. Microorganisms.

[4] Hooper L.V., Midtvedt T., Gordon J.I. (2002). How host-microbial interactions shape the nutrient environment of the mammalian intestine. Annu. Rev. Nutr..

[5] Liu H., Wang J., He T., Becker S., Zhang G., Li D., Ma X. (2018). Butyrate: A Double-Edged Sword for Health?. Adv. Nutr..

[6] Delzenne N.M., Williams C.M. (2002). Prebiotics and lipid metabolism. Curr. Opin. Lipidol..

[7] Li C.J., Elsasser T.H. (2005). Butyrate-induced apoptosis and cell cycle arrest in bovine kidney epithelial cells: Involvement of caspase and proteasome pathways. J Anim. Sci..

[8] Jan G., Belzacq A.S., Haouzi D., Rouault A., Métivier D., Kroemer G., Brenner C. (2002). Propionibacteria induce apoptosis of colorectal carcinoma cells via short-chain fatty acids acting on mitochondria. Cell Death Differ..

[9] Lin Y.G., Vonk R.J., Slooff M.J.H., Kuipers F., Smit M.J. (1995). Differences in propionate-induced inhibition of cholesterol and triacylglycerol synthesis between human and rat hepatocytes in primary culture. Br. J. Nutr..

[10] Nishina P.M., Freedland R.A. (1990). Effects of propionate on lipid biosynthesis in isolated rat hepatocytes. J. Nutr..

[11] Rinninella E., Raoul P., Cintoni M., Franceschi F., Miggiano G.A.D., Gasbarrini A., Mele M.C. (2019). What is the Healthy Gut Microbiota Composition? A Changing Ecosystem across Age, Environment, Diet, and Diseases. Microorganisms.

[12] Van den Abbeele P., Belzer C., Goossens M., Kleerebezem M., De Vos W.M., Thas O., De Weirdt R., Kerckhof F.M., Van de Wiele T. (2013). Butyrate-producing Clostridium cluster XIVa species specifically colonize mucins in an in vitro gut model. ISME J..

[13] Eckburg P.B., Bik E.M., Bernstein C.N., Purdom E., Dethlefsen L., Sargent M., Gill S.R., Nelson K.E., Relman D.A. (2005). Diversity of the human intestinal microbial flora. Science.

[14] Frank D.N., Amand A.L.S., Feldman R.A., Boedeker E.C., Harpaz N., Pace N.R. (2007). Molecular-phylogenetic characterization of microbial community imbalances in human inflammatory bowel diseases. Proc. Natl. Acad. Sci. USA.

[15] Shen X.J., Rawls J.F., Randall T.A., Burcall L., Mpande C., Jenkins N., Jovov B., Abdo Z., Sandler R.S., Keku T.O. (2010). Molecular characterization of mucosal adherent bacteria and associations with colorectal adenomas. Gut Microbes.

[16] Wang Y., Antonopoulos D., Zhu X., Harrell L., Hanan I., Alverdy J., Meyer F., Musch M.W., Young V.B., Chang E.B. (2010). Laser capture microdissection and metagenomic analysis of intact mucosa-associated microbial communities of human colon. Appl. Environ. Microbiol..

[17] Willing B.P., Dicksved J., Halfvarson J., Andersson A.F., Lucio M., Zheng Z., Järnerot G., Tysk C., Jansson J.K., Engstrand L. (2010). A pyrosequencing study in twins shows that gastrointestinal microbial profiles vary with inflammatory bowel disease phenotypes. Gastroenterology.

[18] Hong P.-Y., Croix J.A., Greenberg E., Gaskins H.R., Mackie R.I. (2011). Pyrosequencing-based analysis of the mucosal microbiota in healthy individuals reveals ubiquitous bacterial groups and micro-heterogeneity. PLoS ONE.

[19] Gibson G.R., Hutkins R., Sanders M.E., Prescott S.L., Reimer R.A., Salminen S.J., Scott K., Stanton C., Swanson K.S., Cani P.D. (2017). Expert consensus document: The International Scientific Association for Probiotics and Prebiotics (ISAPP) consensus statement on the definition and scope of prebiotics. Nat. Rev. Gastroenterol. Hepatol..

[20] Minekus M., Alminger M., Alvito P., Balance S., Bohn T., Bourlieu C., Carrière F., Boutrou R., Corredig M., Dupont D. (2014). A standardised static in vitro digestion method suitable for food—An international consensus. Food Funct..

[21] Hodoniczky J., Morris C.A., Rae A.L. (2012). Oral and intestinal digestion of oligosaccharides as potential sweeteners: A systematic evaluation. J. Food Chem..

[22] Sonnenburg E.D., Zheng H., Joglekar P., Higginbottom S.K., Firbank S.J., Bolam D.N., Sonnenburg J.L. (2010). Specificity of polysaccharide use in intestinal bacteroides species determines diet-induced microbiota alterations. Cell.

[23] Hiippala K., Kainulainen V., Suutarinen M., Heini T., Bowers J.R., Jasso-Selles D., Lemmer D., Valentine M., Barnes R., Engelthaler D.M. (2020). Isolation of Anti-Inflammatory and Epithelium Reinforcing Bacteroides and Parabacteroides Spp. from A Healthy Fecal Donor. Nutrients.

[24] Coenen G.J., Bakx E., Verhoef R.P., Schols H.A., Voragen A. (2007). Identification of the connecting linkage between homo- or xylogalacturonan and rhamnogalacturonan type I. Carbohydrate. Polymers.

[25] Wu D., Zheng J., Mao G., Hu W., Ye X., Linhardt R.J., Chen S. (2019). Rethinking the impact of RG-I mainly from fruits and vegetables on dietary health. Crit. Rev. Food Sci. Nutr..

[26] Chakaroun R.M., Massier L., Kovacs P. (2020). Gut Microbiome, Intestinal Permeability, and Tissue Bacteria in Metabolic Disease: Perpetrators or Bystanders?. Nutrients.

[27] Marzorati M., Pinheiro I., Van den Abbeele P., Van de Wiele T., Possemiers S. (2013). An in vitro technology platform to assess host microbiota interactions in the gastrointestinal tract. Agro Food Ind. Hi Tech.

[28] Satsu H., Ishimoto Y., Nakano T., Mochizuki T., Iwanaga T., Shimizu M. (2006). Induction by activated macrophage-like THP-1 cells of apoptotic and necrotic cell death in intestinal epithelial Caco-2 monolayers via tumor necrosis factor-alpha. Exp. Cell Res..

[29] Van den Abbeele P., Taminiau B., Pinheiro I., Duysburgh C., Jacobs H., Pijls L., Marzorati M. (2018). Arabinoxylo-Oligosaccharides and Inulin Impact Inter-Individual Variation on Microbial Metabolism and Composition, Which Immunomodulates Human Cells. J. Agric. Food Chem..

[30] Daguet D., Pinheiro I., Verhelst A., Possemiers S., Marzorati M. (2016). Arabinogalactan and fructooligosaccharides improve the gut barrier function in distinct areas of the colon in the Simulator of the Human Intestinal Microbial Ecosystem. J. Funct. Foods.

[31] Possemiers S., Pinheiro I., Verhelst A., Van den Abbeele P., Maignien L., Laukens D., Reeves S.G., Robinson L.E., Raas T., Schneider Y.-J. (2013). A dried yeast fermentate selectively modulates both the luminal and mucosal gut microbiota and protects against inflammation, as studied in an integrated in vitro approach. J. Agric. Food Chem..

[32] Kleiveland C.R., Verhoeckx K., Cotter P., López-Expósito I., Kleiveland C., Lea T., Mackie A., Requena T., Swiatecka D., Wichers H. (2015). Peripheral Blood Mononuclear Cells. The Impact of Food Bioactives on Health: In vitro and Ex Vivo Models.

[33] Van den Abbeele P., Moens F., Pignataro G., Schnurr J., Ribecco C., Gramenzi A., Marzorati M. (2020). Yeast-Derived Formulations Are Differentially Fermented by the Canine and Feline Microbiome As Assessed in a Novel In Vitro Colonic Fermentation Model. J. Agric. Food Chem..

[34] Jonker D., Fowler P., Albers R., Tzoumaki M.V., van het Hof K.H., Aparicio-Vergara M. (2020). Safety assessment of rhamnogalacturonan-enriched carrot pectin fraction: 90-day oral toxicity study in rats and in vitro genotoxicity studies. Food Chem. Toxicol..

[35] De Weirdt R., Possemiers S., Vermeulen G., Moerdijk-Poortvliet T.C.W., Boschker H.T.S., Verstraete W., Van de Wiele T. (2010). Human faecal microbiota display variable patterns of glycerol metabolism. FEMS Microbiol. Ecol..

[36] Boon N., Top E.M., Verstraete W., Siciliano S.D. (2003). Bioaugmentation as a tool to protect the structure and function of an activated-sludge microbial community against a 3-chloroaniline shock load. Appl. Environ. Microbiol..

[37] Furet J.P., Firmesse O., Gourmelon M., Bridonneau C., Tap J., Mondot S., Doré J., Corthier G. (2009). Comparative assessment of human and farm animal faecal microbiota using real-time quantitative PCR. FEMS Microbiol. Ecol..

[38] Rinttilä T., Kassinen A., Malinen E., Kroqius L., Palva A. (2004). Development of an extensive set of 16S rDNA-targeted primers for quantification of pathogenic and indigenous bacteria in faecal samples by real-time PCR. J. Appl. Microbiol..

[39] Collado M.C., Derrien M., Isolauri E., de Vos W.M., Salminen S. (2007). Intestinal integrity and Akkermansia muciniphila, a mucin-degrading member of the intestinal microbiota present in infants, adults, and the elderly. Appl. Environ. Microbiol..

[40] Guo X., Xia X., Tang R., Zhou J., Zhao H., Wang K. (2008). Development of a real-time PCR method for Firmicutes and Bacteroidetes in faeces and its application to quantify intestinal population of obese and lean pigs. Lett. Appl. Microbiol..

[41] Nakano S., Kobayashi T., Funabiki K., Matsumura A., Nagao Y., Yamada T. (2003). Development of a PCR assay for detection of Enterobacteriaceae in foods. J. Food Prot..

[42] Sokol H., Seksik P., Furet J.P., Firmesse O., Nion-Larmurier I., Beaugerie L., Cosnes J., Corthier G., Marteau P., Doré J. (2009). Low counts of Faecalibacterium prausnitzii in colitis microbiota. Inflamm. Bowel Dis..

[43] Ramirez-Farias C., Slezak K., Fuller Z., Duncan A., Holtrop G., Louis P. (2009). Effect of inulin on the human gut microbiota: Stimulation of Bifidobacterium adolescentis and Faecalibacterium prausnitzii. Br. J. Nutr..

[44] Props R., Kerckhof F.-M., Rubbens P., De Vrieze J., Hernandez Sanabria E., Waegeman W., Monsieurs P., Hammes F., Boon N. (2017). Absolute quantification of microbial taxon abundances. ISME J..

[45] Klindworth A., Pruesse E., Schweer T., Peplies J., Quast C., Horn M., Glöckner F.O. (2013). Evaluation of general 16S ribosomal RNA gene PCR primers for classical and next-generation sequencing-based diversity studies. Nucleic Acids Res..

[46] Schloss P.D., Westcott S.L. (2011). Assessing and improving methods used in operational taxonomic unit-based approaches for 16S rRNA gene sequence analysis. Appl. Environ. Microbiol..

[47] Kozich J.J., Westcott S.L., Baxter N.T., Highlander S.K., Schloss P.D. (2013). Development of a dual-index sequencing strategy and curation pipeline for analyzing amplicon sequence data on MiSeq Illumina sequencing platform. Appl. Environ. Microbiol..

[48] Wang Q., Garrity G.M., Tiedje J.M., Cole J.R. (2007). Naive Bayesian classifier for rapid assignment of rRNA sequences into the new bacterial taxonomy. Appl. Environ. Microbiol..

[49] Cole J.R., Wang Q., Cardenas E., Fish J., Chai B., Farris R.J., Kulam-Syed-Mohideen A.S., McGarrell D.M., Marsh T., Garrity G.M. (2009). The ribosomal database project: Improved alignments and new tools for rRNA analysis. Nucleic Acids Res.

[50] Louis P., Flint H.J. (2017). Formation of propionate and butyrate by the human colonic microbiota. Environ. Microbiol..

[51] Hamer H.M., De Preter V., Windey K., Verbeke K. (2012). Functional analysis of colonic bacterial metabolism: Relevant to health. Am. J. Physiol. Liver Physiol..

[52] Scott K.P., Gratz S.W., Sheridan P.O., Flint H.J., Duncan S.H. (2013). The influence of diet on the gut microbiota. Pharmacol. Res..

[53] Kempski J., Brockmann L., Gagliani N., Huber S. (2017). TH17 Cell and Epithelial Cell Crosstalk during Inflammatory Bowel Disease and Carcinogenesis. Front Immunol..

[54] Kurita-Ochiai T., Fukushima K., Ochiai K. (1999). Lipopolysaccharide stimulates butyric acid-induced apoptosis in human peripheral blood mononuclear cells. Infect. Immun..

[55] Weber T.E., Kerr B.J. (2006). Butyrate differentially regulates cytokines and proliferation in porcine peripheral blood mononuclear cells. Vet. Immunol. Immunopathol..

[56] Bonnema A.L., Kolberg L.W., Thomas W., Slavin J.L. (2010). Gastrointestinal tolerance of chicory inulin products. J. Am. Diet. Assoc..

[57] Timm D.A., Stewart M.L., Hospattankar A., Slavin J.L. (2010). Wheat Dextrin, Psyllium, and Inulin Produce Distinct FermentationPatterns, GasVolumes, andShort-Chain Fatty AcidProfiles InVitro. J. Med. Food.

[58] Carlson J.L., Erickson J.M., Hess J.M., Gould T.J., Slavin J.L. (2017). Prebiotic Dietary Fiber and Gut Health: Comparing the in Vitro Fermentations of Beta-Glucan, Inulin and Xylooligosaccharide. Nutrients.

[59] Belenguer A., Duncan S.H., Calder A.G., Holtrop G., Louis P., Lobley G.E., Flint H.J. (2006). Two Routes of Metabolic Cross-Feeding between Bifidobacterium adolescentis and Butyrate-Producing Anaerobes from the Human Gut. Appl. Environ. Microbiol..

[60] De Vuyst L., Moens F., Selak M., Rivière A., Leroy F. (2014). Summer Meeting 2013: Growth and physiology of bifidobacteria. J. Appl. Microbiol..

[61] Milani C., Lugli G.A., Duranti S., Turroni F., Mancabelli L., Ferrario C., Mangifesta M., Hevia A., Viappiani A., Scholz M. (2015). Bifidobacteria exhibit social behavior through carbohydrate resource sharing in the gut. Sci. Rep..

[62] Moens F., Weckx S., De Vuyst L. (2016). Bifidobacterial inulin-type fructan degradation capacity determines cross-feeding interactions between bifidobacteria and Faecalibacterium prausnitzii. Int. J. Food Microbiol..

[63] Fukuda S., Toh H., Hase K., Oshima K., Nakanishi Y., Yoshimura K., Tobe T., Clarke J.M., Topping D.L., Suzuki T. (2011). Bifidobacteria can protect from enteropathogenic infection through production of acetate. Nature.

[64] Srutkova D., Schwarzer M., Hudcovic T., Zakostelska Z., Drab V., Spanova A., Rittich B., Kozakova H., Schabussova I. (2015). Bifidobacterium longum CCM 7952 Promotes Epithelial Barrier Function and Prevents Acute DSS-Induced Colitis in Strictly Strain-Specific Manner. PLoS ONE.

[65] Furrie E., Macfarlane S., Kennedy A., Cummings J.H., Walsh S.V., O’neil D.A., Macfarlane G.T. (2005). Synbiotic therapy (Bifidobacterium longum/Synergy 1) initiates resolution of inflammation in patients with active ulcerative colitis: A randomised controlled pilot trial. Gut.

[66] Yu R., Zuo F., Ma H., Chen S. (2019). Exopolysaccharide-Producing Bifidobacterium adolescentis Strains with Similar Adhesion Property Induce Differential Regulation of Inflammatory Immune Response in Treg/Th17 Axis of DSS-Colitis Mice. Nutrients.

[67] Hosseini E., Grootaert C., Verstraete W., Van de Wiele T. (2011). Propionate as a health-promoting microbial metabolite in the human gut. Nutr. Rev..

[68] Yoshida N., Emoto T., Yamashita T., Watanabe H., Hayashi T., Tabata T., Hoshi N., Hatano N., Ozawa G., Sasaki N. (2018). Bacteroides vulgatus and Bacteroides dorei Reduce Gut Microbial Lipopolysaccharide Production and Inhibit Atherosclerosis. Circulation.

[69] Ihekweazu F.D., Fofanova T.Y., Queliza K., Nagy-Szakal D., Stewart C.J., Engevik M.A., Hulten K.G., Tatevian N., Graham D.Y., Versalovic J. (2019). Bacteroides ovatus ATCC 8483 monotherapy is superior to traditional fecal transplant and multi-strain bacteriotherapy in a murine colitis model. Gut Microbes.

[70] Morotomi M., Nagai F., Sakon H., Tanaka R. (2008). Dialister succinatiphilus sp. nov. and Barnesiella intestinihominis sp. nov., isolated from human faeces. Int. J. Syst. Evol. Microbiol..

[71] Sears C.L. (2009). Enterotoxigenic Bacteroides fragilis: A rogue among symbiotes. Clin. Microbiol. Rev..

[72] Wei B., Dalwadi H., Gordon L.K., Landers C., Bruckner D., Targan S.R., Braun J. (2001). Molecular cloning of a Bacteroides caccae TonB-linked outer membrane protein identified by an inflammatory bowel disease marker antibody. Infect. Immun..

[73] Patterson A.M., Mulder I.E., Travis A.J., Lan A., Cerf-Bensussan N., Gaboriau-Routhiau V., Garden K., Logan E., Delday M.I., Coutts A.G.P. (2017). Human Gut Symbiont Roseburia hominis Promotes and Regulates Innate Immunity. Front. Immunol..

[74] Machiels K., Joossens M., Sabino J., De Preter V., Arijs I., Eeckhaut V., Ballet V., Claes K., Van Immerseel F., Verbeke K. (2014). A decrease of the butyrate-producing species Roseburia hominis and Faecalibacterium prausnitzii defines dysbiosis in patients with ulcerative colitis. Gut.

[75] Tilg H., Danese S. (2014). Roseburia hominis: A novel guilty player in ulcerative colitis pathogenesis?. Gut.

[76] Qiu X., Zhang M., Yang X., Hong N., Yu C. (2013). Faecalibacterium prausnitzii upregulates regulatory T cells and anti-inflammatory cytokines in treating TNBS-induced colitis. J. Crohns Colitis.

[77] Udayappan S., Manneras-Holm L., Chaplin-Scott A., Belzer C., Herrema H., Dallinga-Thie G.M., Duncan S.H., Stroes E.S.G., Groen A.K., Flint H.J. (2016). Oral treatment with Eubacterium hallii improves insulin sensitivity in db/db mice. NPJ Biofilms Microbiomes.

[78] Murai M., Turovskaya O., Kim G., Madan R., Karp C.L., Cheroutre H., Kronenberg M. (2009). Interleukin 10 acts on regulatory T cells to maintain expression of the transcription factor Foxp3 and suppressive function in mice with colitis. Nat. Immunol..

[79] Kumar P., Monin L., Castillo P., Elsegeiny W., Horne W., Eddens T., Vikram A., Good M., Schoenborn A.A., Bibby K. (2016). Intestinal Interleukin-17 Receptor Signaling Mediates Reciprocal Control of the Gut Microbiota and Autoimmune Inflammation. Immunity..

[80] Chen M.L., Sundrud M.S. (2016). Cytokine Networks and T-Cell Subsets in Inflammatory Bowel Diseases. Inflamm. Bowel Dis..

[81] Wang Y.H. (2016). Developing food allergy: A potential immunologic pathway linking skin barrier to gut. F1000Research.

[82] Clark D.P. (1989). The fermentation pathways of Escherichia coli. FEMS Microbiol. Rev..

[83] Rey F.E., Gonzalez M.D., Cheng J., Wu M., Ahern P.P., Gordon J.I. (2013). Metabolic niche of a prominent sulfate-reducing human gut bacterium. Proc. Natl. Acad. Sci. USA.

