# Supplementary material for: A Novel Non-Digestible, Carrot-Derived Polysaccharide (cRG-I) Selectively Modulates the Human Gut Microbiota while Promoting Gut Barrier Integrity: An Integrated In Vitro Approach
Source: Nutrients. 2020 Jun 29;12(7):1917. doi: 10.3390/nu12071917 (PMC7400138; doi:10.3390/nu12071917)
Supplement: Supplementary file 1 [file nutrients-12-01917-s001.zip › supplementary files-1/Supplementary Figure S1 + legend. V2.docx]

Supplementary Figure S1

**Figure S1.** Effect of Sodium butyrate (NaB) on transepithelial electrical resistance (TEER) and cytokine production in a Caco-2/PBMC co-culture system. Caco-2 cells, cultured 14 days on transwell inserts, were placed on top of pokeweed mitogen (PWM+)-activated PBMCs and incubated for 48h in absence or present of 5 mM NaB at the apical side. Average (±SEM) TEER of the Caco-2 monolayers (A) and concentration of secreted interferon (IFN)γ (B), interleukin (IL)-17A (C), IL-21 (D), IL-22 (E), IL-4 (F), IL-9 (G) and IL-10 (H) in the basolateral medium upon 48h of incubation are shown (n=3). Statistically significant differences to PWM+ are represented by (*). (*) = p<0.05, (**) = p<0.01, (***) = p<0.001 and (****) = p<0.0001.
