# Supplementary material for: A Novel Non-Digestible, Carrot-Derived Polysaccharide (cRG-I) Selectively Modulates the Human Gut Microbiota while Promoting Gut Barrier Integrity: An Integrated In Vitro Approach
Source: Nutrients. 2020 Jun 29;12(7):1917. doi: 10.3390/nu12071917 (PMC7400138; doi:10.3390/nu12071917)
Supplement: Supplementary file 1 [file nutrients-12-01917-s001.zip › supplementary files-1/Supplementary-tables.docx]

| **Phylum** | **Family** | **OTU number** | **Species to which OTU is related** | **Inoculum** | **L** | | | **M** | | | | | |
| --- | --- | --- | --- | --- | --- | --- | --- | --- | --- | --- | --- | --- | --- |
|  |  |  |  |  | **lumen** | | | **lumen** | | | **mucus** | | |
|  |  |  |  |  | **blank** | **cRG-I** | **cRG-I+LMWC** | **blank** | **cRG-I** | **cRG-I+LMWC** | **blank** | **cRG-I** | **cRG-I+LMWC** |
| Actinobacteria | Bifidobacteriaceae | Otu0007 | *Bifidobacterium longum* | 0.3% | 0.3% | **2.7%** | **2.1%** | 0.2% | **3.0%** | **1.7%** | 0.2% | **6.1%** | 1.1% |
|  |  | Otu0021 | *Bifidobacterium adolescentis* | 1.1% | 0.2% | **2.9%** | **0.8%** | 0.1% | **1.9%** | **0.7%** | 0.0% | **0.2%** | 0.1% |
|  | Coriobacteriaceae | Otu0015 | *Collinsella aerofaciens* | 12.0% | 0.1% | **0.0%** | 0.1% | 0.1% | **0.0%** | 0.1% | 0.0% | 0.0% | 0.0% |
| Bacteroidetes | Bacteroidaceae | Otu0013 | *Bacteroides caccae* | 0.3% | 1.2% | **0.6%** | **0.7%** | 1.5% | **0.7%** | **0.7%** | 5.1% | **0.6%** | **0.7%** |
|  |  | Otu0002 | *Bacteroides dorei* | 2.5% | 9.0% | **34.4%** | **38.3%** | 8.1% | **37.2%** | **29.8%** | 1.9% | **4.9%** | *4.3%* |
|  |  | Otu0011 | *Bacteroides fragilis* | 0.1% | 0.9% | **0.4%** | **0.6%** | 1.4% | **0.5%** | **0.6%** | 9.1% | **0.8%** | **0.7%** |
|  |  | Otu0022 | *Bacteroides ovatus* | 0.0% | 0.3% | **2.5%** | **1.2%** | 0.3% | **2.0%** | **1.0%** | 0.1% | 0.1% | 0.2% |
|  |  | Otu0006 | *Bacteroides plebeius* | 0.3% | 0.0% | **2.9%** | **3.7%** | 0.1% | **4.1%** | **3.3%** | 0.6% | **1.8%** | **1.5%** |
|  |  | Otu0016 | *Bacteroides thethaiotaomicron* | 0.0% | 1.0% | 1.2% | **1.3%** | 1.2% | 1.4% | 1.0% | 1.4% | **0.3%** | **0.5%** |
|  |  | Otu0017 | *Bacteroides uniformis* | 1.4% | 2.3% | **0.9%** | **0.8%** | 1.9% | **0.9%** | **0.7%** | 0.4% | 0.1% | 0.2% |
|  |  | Otu0018 | *Bacteroides xylanisolvens* | 0.1% | 0.3% | **2.6%** | **0.9%** | 0.3% | **2.6%** | **0.8%** | 0.5% | 0.4% | **0.3%** |
| Firmicutes | Clostridiaceae cluster I | Otu0014 | *Clostridium butyricum* | 0.0% | 0.0% | 0.0% | 0.0% | 0.0% | 0.0% | 0.0% | *10.3%* | 0.0% | 0.0% |
|  |  | Otu0010 | *Clostridium tertium* | 0.0% | 0.0% | 0.0% | 0.0% | 0.1% | **0.0%** | **0.0%** | 14.4% | **0.9%** | **0.4%** |
|  |  | Otu0008 | *Clostridium paraputrificum* | 0.0% | 0.0% | 0.0% | 0.0% | 0.0% | 0.0% | 0.0% | 16.9% | **1.2%** | **1.4%** |
|  | Erysipelotrichaceae | Otu0086 | *Clostridium innocuum* | 0.0% | 0.0% | **0.1%** | 0.0% | 0.0% | **0.1%** | **0.1%** | 0.0% | **0.2%** | 0.0% |
|  | Lachnospiraceae | Otu0027 | *Blautia wexlerae* | 3.6% | 0.7% | **0.3%** | **0.3%** | 0.4% | **0.1%** | **0.2%** | 0.1% | 0.1% | **0.0%** |
|  |  | Otu0029 | *Butyrate producing bacterium SS3/4-GM2/1* | 0.2% | 1.1% | **0.2%** | 1.4% | 1.0% | **0.2%** | 0.6% | 0.1% | 0.0% | 0.1% |
|  |  | Otu0030 | *Eisenbergiella tayi* | 0.0% | 0.4% | **0.8%** | 0.5% | 0.5% | **0.7%** | 0.4% | 0.8% | 0.3% | 0.2% |
|  |  | Otu0035 | *Lachnospiraceae GAM79* | 0.1% | 0.0% | 0.0% | 0.0% | 0.0% | 0.0% | 0.0% | 4.1% | **0.0%** | **0.4%** |
|  |  | Otu0003 | *Roseburia hominis* | 0.8% | 0.0% | 0.0% | 0.0% | 0.0% | 0.0% | 0.0% | 9.8% | **64.3%** | **74.2%** |
|  |  | Otu0024 | *Ruminococcus faecis* | 2.7% | 0.7% | 0.9% | **0.3%** | 0.6% | **0.3%** | **0.2%** | 0.4% | 0.6% | **0.0%** |
|  |  | Otu0019 | *Ruminococcus lactaris* | 0.2% | 0.2% | **0.0%** | **0.0%** | 0.6% | **0.1%** | **0.1%** | *5.4%* | 2.2% | 0.8% |
|  |  | Otu0012 | *Ruminococcus torques* | 0.0% | 0.7% | **0.2%** | **0.1%** | 1.6% | **0.3%** | **0.2%** | *4.4%* | 3.7% | 1.7% |
|  |  | Otu0038 | *<97% similarity to known species* | 0.1% | 2.5% | **0.3%** | **0.0%** | 1.3% | **0.0%** | **0.0%** | 0.1% | 0.0% | 0.0% |
|  | Ruminococcaceae | Otu0083 | *Faecalibacterium prausnitzii* | 0.1% | 0.0% | **0.1%** | 0.0% | 0.0% | **0.1%** | 0.0% | 0.0% | *0.3%* | 0.0% |
|  |  | Otu0005 | *Faecalibacterium prausnitzii* | 15.6% | *0.1%* | **2.6%** | **1.0%** | 0.2% | **2.9%** | **1.5%** | 0.3% | *1.3%* | 0.5% |
|  |  | Otu0020 | *Gemmiger formicilis* | 4.9% | 0.6% | **1.3%** | **0.4%** | 0.6% | 0.9% | 0.5% | 0.1% | 0.1% | 0.0% |
|  |  | Otu0066 | *<90% similarity to known species* | 0.1% | 0.0% | **0.9%** | 0.0% | 0.0% | **0.1%** | 0.0% | 0.0% | 0.0% | 0.0% |
|  | Streptococcaceae | Otu0026 | *Streptococcus anginosus* | 0.0% | 0.0% | **0.1%** | **2.1%** | 0.0% | 0.0% | **2.0%** | 0.1% | *1.0%* | **0.5%** |
|  | Veillonellaceae | Otu0009 | *Dialister succinatiphilus* | 4.2% | 1.7% | **3.2%** | 1.5% | 1.5% | **2.3%** | **1.9%** | 0.2% | 0.2% | 0.1% |
| Proteobacteria | Enterobacteriaceae | Otu0001 | *Escherichia coli - Shigella flexneri* | 0.1% | 46.5% | **24.4%** | **34.4%** | 37.7% | **22.5%** | 37.8% | 2.8% | 2.7% | *5.1%* |
| Verrucomicrobia | Akkermansiaceae | Otu0004 | *Akkermansia muciniphila* | 3.1% | 3.1% | **0.4%** | **0.8%** | 13.4% | **5.5%** | **5.8%** | 0.6% | 0.2% | 0.4% |

Legend: **Supplementary table S1.** Effect of fermentation of cRG-I and cRG-I+LMWC on microbial community composition at OTU level in short-term colonic batch simulations in absence or presence of a mucosal compartment. Average proportional abundance of the selected OTUs in the original (diluted) inoculum (in) and after 48h of incubation upon dosing of cRG-I and cRG-I +LMWC to the gut microbiota of a healthy donor in incubations containing only a luminal (L) or additionally also a mucosal (M) environment versus a blank control (n=3). Statistically significant differences between the blank and treatments are indicated in bold (p<0.05). Upon reaching statistically significant differences; highest values are underlined. Values indicated in italics are strongly increased; although not significant.

|  | **L** | | | **M** | | | | | |
| --- | --- | --- | --- | --- | --- | --- | --- | --- | --- |
|  | **lumen** | | | **lumen** | | | **mucus** | | |
| **Family/genus/species** | **blank** | **cRG-I** | **cRG-I+LMWC** | **blank** | **cRG-I** | **cRG-I+LMWC** | **blank** | **cRG-I** | **cRG-I+LMWC** |
| *Lactobacilli* | 5.11 | 4.92 | 5.18 | 5.15 | 4.87 | 5.11 | <LOQ | <LOQ | <LOG |
| *Bifidobacteria* | 8.14 | **8.96** | **8.89** | 8.13 | **9.08** | **8.97** | 7.03 | **8.14** | **7.67** |
| *Akkermansia muciniphila* | 8.44 | 8.19 | 8.48 | 9.06 | 9.11 | 9.15 | 7.13 | 6.77 | 7.21 |
| *Bacteroidetes* | 9.23 | **9.91** | **9.93** | 9.34 | **10.07** | **9,91** | 8.59 | **8.27** | **8.27** |
| *Enterobacteriaceae* | 10.21 | 10.18 | **10.46** | 10.29 | 10.27 | **10.57** | 8.55 | 8.58 | **8.91** |
| *Faecalibacterium prausnitzii* | 9.53 | **10.00** | **9.68** | 9.62 | **9.88** | 9.71 | 8.05 | 8.24 | 7.93 |
| *Roseburia* | 7.45 | 7.30 | 7.41 | 7.45 | 7.43 | **7.57** | 8.15 | **9.07** | **9.19** |
| *Eubacterium rectale/Clostridium coccoides* | 9.42 | **9.49** | 9.43 | 9.50 | 9.51 | 9.46 | 8.95 | **9.30** | **9.38** |
| *Eubacterium hallii* | 7.82 | 7.90 | 7.82 | 7.81 | 7.89 | **8.06** | 5.59 | **6.19** | **6.17** |

Legend: **Supplementary table S2.** Effect of fermentation of cRG-I and cRG-I +LMWC on selected bacterial groups in short-term colonic batch simulations in absence or presence of a mucosal compartment as assessed through qPCR. Average absolute abundance of Lactobacilli, Bifidobacteria, Akkermansia muciniphila, Bacteroidetes, Enterobacteriaceae, Faecalibacterium prausnitzii, Roseburia, Eubacterium rectale/Clostridum coccoides and Eubacterium hallii after 48h of incubation upon dosing of cRG-I and cRG-I +LMWC to the gut microbiota of a healthy donor during incubations containing only a luminal (L) or additionally also a mucosal (M) environment versus a blank control (n=3). Statistically significant differences between the blank and treatments are indicated in bold (p<0.05). Upon reaching statistically significant differences; highest values are underlined.
